# The role of leukotriene modifying agent treatment in neuropsychiatric events of elderly asthma patients: a nested case control study

**DOI:** 10.1186/s40733-021-00070-4

**Published:** 2021-03-17

**Authors:** Sang Oh Kang, Kyung Hyun Min, Hyun Jeong Kim, Tae Hyeok Kim, Woorim Kim, Kyung Eun Lee

**Affiliations:** College of Pharmacy, Chungbuk National University, 660-1 Yeonje-ri, Osong-eup, Heungdeok-gu, Cheongju-si, 28160 Republic of Korea

**Keywords:** Leukotriene modifying agent, Neuropsychiatric events, National health insurance service database, South Korea, Asthma, Nested case-control study

## Abstract

**Background:**

In March 2020, the US Food and Drug Administration decided that the dangers related to neuropsychiatric events (NPEs) of montelukast, one of the leukotriene modifying agents (LTMAs), should be communicated through ‘boxed warning’. In case of NPEs, the prevalence has been the highest in elderly people. Because the characteristics of the elderly such as old age itself can act as risk factors. Therefore, an investigation on safety of LTMAs related to NPEs in elderly using LTMAs is needed.

**Method:**

A nested case-control study using an elderly sample cohort from the Korean National Health Insurance Service database was used. The asthma cohort included asthma patients newly diagnosed between 2003 and 2013. Within the asthma cohort, the case group was defined as patients who were diagnosed with NPEs. Among patients who had never been diagnosed with NPEs, the control group was selected by matching 1:1 by propensity score. Patients who were prescribed LTMAs for 1 year prior to index date were defined as the exposure group. The logistic regression model was used to measure the effect of LTMAs on NPEs.

**Results:**

We identified 141,165 patients with newly diagnosed asthma, and selected 31,992 patients per each case and control group. Exposure to LTMAs significantly increased the risk of overall NPEs about in comparison with the absence of exposure (crude odds ratio [OR] 1.58, 95% CI 1.50–1.68). After adjusting for confounding factors, the overall NPEs risk increased (adjusted OR, 1.67, 95% CI 1.58–1.78).

**Conclusion:**

This study suggests that elderly asthma patients prescribed LTMAs had a higher risk of NPEs than patients who were not treated with LTMAs. Therefore, clinicians should be aware of the potential risks of LTMAs.

## Background

Asthma is the most common chronic airway disease that affected approximately 358 million patients in 2015 globally [1]. It is characterized by chronic airway inflammation and recurring symptoms such as wheezing, dyspnea, chest-tightness, and coughing [2]. Asthma is typically managed with a combination of long-term maintenance therapy (controller medications) and short-term therapy for the relief of acute asthma symptoms (reliever medications) [3]. Leukotriene modifying agents (LTMAs), including montelukast, pranlukast and zafirlukast, are one of the maintenance medications [4]. These agents function by chemically modifying and inhibiting an inflammatory mediator called leukotriene, which causes long-lasting bronchoconstriction and increases mucus production. These agents can be used to improve lung function and decrease the requirement for β-adrenergic agonists, resulting in significant symptom control [5].

In 2009, the US Food and Drug Administration (FDA) announced a label change for montelukast to include a warning regarding neuropsychiatric events (NPEs) under the “Precautions” section. This label change was triggered by post-marketing case reports to the FDA Adverse Event Reporting System. Specifically, patients prescribed montelukast reported episodes of depression, anxiety, sleep disturbance, aggression/agitation, suicidal ideation, suicide attempts, and/or completed suicide [6]. FDA continued to receive case reports of mental health side effects associated with montelukast use and conducted an observational study using data from its Sentinel System. Consistent with the prior evaluations of FDA, a wide variety of mental health side effects were found (including completed suicides). Some occurred during montelukast treatment and resolved after stopping the medicine. Thus, in March 2020, the FDA announced that montelukast required a Black Boxed Warning, their most prominent warning [7].

In Korea, there were nearly 2.22 milllon of asthma patients in 2010 and the prevalence of asthma was high, especially in the population aged over 60 years. The percentage of LTMAs among prescribed anti-asthma drugs has been growing (26.2% in 2002 and 63.1% in 2015 in uncontrolled asthma; 46.4% in 2002 and 76.4% in 2015 in severe asthma) [8]. Also, the data suggest increasing use of LTMAs in elderly asthma patients.

The elderly are more vulnerable to NPEs than the general population. As well as, the prevalence of NPEs such as depression and sleep disorder is higher in the elderly [9, 10]. Despite the FDA regulation on using montelukast, studies including elderly asthma patients or examining the association of NPEs in other LTMAs are scarce.

Therefore, we aimed to investigate whether LTMA use was associated with the risk of NPE development and also to examine if this is a class effect of LTMAs, especially in elderly asthma patients by using large population database.

## Methods

### Data source

This is a nested case-control study using the Korean National Health Insurance Service (NHIS) elderly cohort data. NHIS database contains medical claims data for more than 99% of the South Korean population and has long-period of follow up. It contains individual beneficiary and healthcare service information including diagnoses, procedures, and prescriptions. The information on diagnoses were coded according to the International Classification of Diseases, Tenth Revision (ICD-10).

The NHIS elderly database contains stratified random samples of claims data, and the size of the samples was calculated and data were extracted on a yearly basis to improve the representativeness of the socio-demographic characteristics, diagnosis, and healthcare services including prescription drugs for Korean patients. The National Health Insurance program in Korea has provided a comprehensive elderly cohort database that supports researches on analysis of the risk factors of prevalent diseases and prognosis in elderly patients.

### Study samples and design

#### Nested case-control study

A nested case control study was used to investigate the association between LTMA treatment and diagnosis of NPEs. ‘Nested’ means that subjects included in a defined cohort become baseline patients for the selection of the case and control. In a retrospective nested case-control study such as this study, a case, affected by the disease, is matched with one or more individuals not affected by the disease, the controls [11].

#### Asthma subjects

The asthma subjects included elderly cohort (aged over 60) who were newly diagnosed with asthma (ICD-10, J45) or status asthmaticus (ICD-10, J46) between 2003 and 2013 and had been prescribed an asthma disease controller [12] (inhalers: budesonide, ciclesonide, fluticasone, beclomethasone, beclomethasone/formoterol, budesonide/formoterol, fluticasone/formoterol, fluticasone/vilanterol, fluticasone/salmeterol, tablets: bambuterol, salbutamol, theophylline, aminophylline, doxofylline, montelukast, zafirlukast, pranlukast).

#### Case and control group definition

Among asthma subjects, the case group was defined as patients who were diagnosed with NPEs. The NPEs included mood disorder, sleep disorder, anxiety disorder, personality disorder, substance-related disorder, agitation, schizophrenia and self-harm disease. These NPEs were in accordance with neuropsychiatric diagnosis mentioned in the montelukast product label [13]. Index date of the case group was defined as the date of NPE diagnosis. Patients who were diagnosed with NPEs before asthma diagnosis were excluded from the case group.

**Fig. 1:**
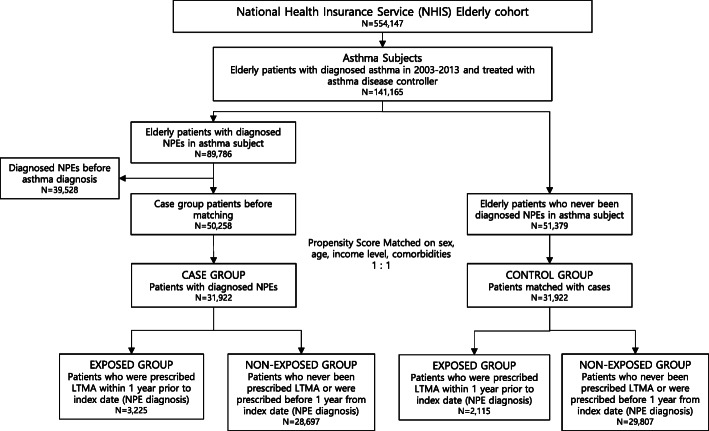
Selection of the case and control group, and exposed and non-exposed groups. Abbreviation: NPE Neuropsychiatric Event

Control group was defined as patients who had never been diagnosed with NPEs and was matched by propensity score considering sex, age, income level and comorbidities. The index date of the control group was selected as a random date between the patient’s first and last diagnosis date of any diseases in the NHIS database (December 31th, 2013). (Fig. 1).

#### Exposure to LTMAs

Patients who were prescribed LTMAs including montelukast, pranlukast or zafirlukast during asthma drug exposure period were defined as the exposure group. The drug exposure period was defined as 1 year before the index date.

“Recency of exposure” was the number of days between the last day of LTMA exposure in the drug exposure period and the index date, and was categorized into 1–60 days, 61–120 days, and 121–365 days. These recency of exposure subgroups were named group 1, group 2, and group 3, respectively.

“Duration of exposure” was defined as the total days of LTMA prescription in the drug exposure period and was categorized into 1–30 days, 31–120 days and greater than 120 days. These exposure duration subgroups were named as group 4, group 5 and group 6, respectively.

#### Variables for adjusting confounders

The following variables were considered when subjects were enrolled. The baseline demographic variables included age, sex, income level, comorbidities, and Charlson Comorbidity Index (CCI). CCI was used to calculate the severity of health status [14] and to consider the effect of comorbidities on NPE development. CCI includes of 15 categories of diseases that can predict the 1-year death rate: myocardial infarction, congestive heart failure, peripheral vascular disease, cerebrovascular disease, dementia, chronic pulmonary disease, rheumatologic disease, peptic ulcer disease, liver disease, diabetes mellitus, hemiplegia or paraplegia, renal disease, malignancies including metastatic solid tumors, and AIDS. Additionally, hypertension, dyslipidemia, arrhythmia and Parkinson’s disease were included as covariates and because there is evidences of their association with NPEs [15, 16].

### Statistical analysis

A chi-square test was performed for categorical variables and a two-sample *t*-test was performed for continuous variables to evaluate the difference between the case and control groups. The logistic regression model was used to measure the effect of LTMAs on NPEs in two ways. Model 1 was logistic regression conducted with only LTMA exposure. Model 2 was adjusted regression with demographic covariates.

First, we categorized regression by the type of NPEs (overall NPEs, sleep disorder, mood disorder, anxiety disorder). Second, we categorized overall NPE regression into three types by sex (total, male, female). Third, we categorized four types of NPE regression into recency of exposure and treatment duration. All data analyses were performed using SAS version 9.4 (SAS Institute Inc. Cary, NC, USA) and statistical significance was inferred at a two-sided *p*-value of < 0.05.

## Results

### Baseline characteristics of the case and control groups

Among 554,147 elderly patients, we identified 141,165 patients who were newly diagnosed with asthma and were prescribed an anti-asthmatic drug between 2003 and 2013. In this senior asthma cohort, 31,992 patients diagnosed with NPEs were selected as the case group.

New-onset NPEs occurred more often in females (58.6% in the case and control groups) and nearly 50% of patients were in their 70s (47.7% in the case group; 48.4% in the control group). Exposure to LTMAs was more common in the case group (3225 cases, 10.1%) than in the control group (2115 cases, 6.6%). Most of the baseline characteristics were well balanced between the two groups. There were no significant differences in age, sex, income level and most comorbidities, although several comorbidities (dementia, hypertension, peripheral vascular disease and chronic pulmonary disease) had a significant association with NPE development between the two groups. (Table 1).

**Table 1:** Baseline demographic characteristics

	CASE group (NPE development)	CONTROL group (No NPE development)	*P* value
Sex			
Male	13,206 (41.4%)	13,221 (41.4%)	0.90
Female	18,716 (58.6%)	18,701 (58.6%)	
Age, mean (SD)	72.7 (6.6)	72.6 (6.6)	0.11
Age group			
60s	11,483 (36.0%)	11,459 (35.9%)	0.10
70s	15,227 (47.7%)	15,440 (48.4%)	
80s	4824 (15.1%)	4619 (14.5%)	
90s	388 (1.2%)	404 (1.3%)	
Income level			
Q0-Q2	8248 (25.8%)	8038 (25.2%)	0.11
Q3-Q5	5465 (17.1%)	5643 (17.7%)	
Q6-Q8	8046 (25.2%)	8117 (25.4%)	
Q9-Q10	10,163 (31.8%)	10,124 (31.7%)	
LTMA use			
Yes	3225 (10.1%)	2115 (6.6%)	< 0.01
No	28,697 (89.9%)	29,807 (93.4%)	
Comorbidities			
CCI score, mean (SD)	2.52 (2.14)	2.51 (2.19)	0.55
Group 1 (0)	3320 (10.4%)	3489 (10.9%)	0.06
Group 2 (2 ≥ cci ≥1)	16,161 (50.6%)	16,173 (50.7%)	
Group 3 (cci ≥ 3)	12,441 (39.0%)	12,260 (38.4%)	
Myocardial infarction			
Yes	971 (3.0%)	935 (2.9%)	0.40
No	30,951 (97.0%)	30,987 (97.1%)	
Congestive heart failure			
Yes	3610 (11.3%)	3560 (11.1%)	0.53
No	28,312 (88.7%)	28,362 (88.9%)	
Arrhythmia			
Yes	2131 (6.7%)	2070 (6.5%)	0.33
No	29,791 (93.3%)	29,852 (93.5%)	
Hypertension			
Yes	19,563 (61.3%)	19,882 (62.3%)	< 0.01
No	12,359 (38.7%)	12,040 (37.7%)	
Dyslipidemia			
Yes	9630 (30.2%)	9510 (29.8%)	0.30
No	22,292 (69.8%)	22,412 (70.2%)	
Peripheral vascular disease			
Yes	5080 (15.9%)	4892 (15.3%)	0.04
No	26,842 (84.1%)	27,030 (84.7%)	
Cerebrovascular disease			
Yes	5289 (16.6%)	5108 (16.0%)	0.05
No	26,633 (83.4%)	26,814 (84.0%)	
Dementia			
Yes	1745 (5.5%)	1495 (4.7%)	< 0.01
No	30,177 (94.5%)	30,427 (95.3%)	
Parkinson’s disease			
Yes	422 (1.3%)	374 (1.2%)	0.09
No	31,500 (98.7%)	31,548 (98.8%)	
Chronic pulmonary disease			
Yes	19,022 (59.6%)	19,449 (60.9%)	< 0.01
No	12,900 (40.4%)	12,473 (39.1%)	
Rheumatoid arthritis			
Yes	1779 (5.6%)	1789 (5.6%)	0.86
No	30,143 (94.4%)	30,133 (94.4%)	
Peptic ulcer disease			
Yes	10,512 (32.9%)	10,402 (32.6%)	0.35
No	21,410 (67.1%)	21,520 (67.4%)	
Liver disease			
Yes	6378 (20.0%)	6263 (19.6%)	0.25
No	25,544 (80.0%)	25,659 (80.4%)	
Diabetes mellitus			
Yes	8547 (26.8%)	8434 (26.4%)	0.31
No	23,375 (73.2%)	23,488 (73.6%)	
Hemiplegia or Paraplegia			
Yes	677 (2.1%)	640 (2.0%)	0.30
No	31,245 (97.9%)	31,282 (98.0%)	
Renal disease			
Yes	644 (2.0%)	622 (2.0%)	0.53
No	31,278 (98.0%)	31,300 (98.0%)	
Malignancy			
Yes	2915 (9.1%)	2938 (9.2%)	0.75
No	29,007 (90.9%)	28,984 (90.8%)	
HIV			
Yes	3 (0.01%)	2 (0.02)	0.65
No	31,919 (99.9%)	31,920 (99.99%)	

*Abbreviations*: *NPE* Neuropsyhciatric event, *SD* Standard Deviation, *CCI* Charlson Comorbidity Index, *LTMA* Leukotriene Modifying Agent, *HIV* Human Immunodeficiency Virus

Chi-square test was used for categorical variables, *t*-test was used for numerical variables.

The most frequently prescribed LTMA was montelukast (1788/3225, 55.4% in the case group; 1243/2115, 58.8% in the control group), followed by pranlukast (1409/3225, 43.7% in the case group; 853/2115, 40.3% in the control group), and zafirlukast (28/3225, 0.9% in the case group; 19/2115, 0.9% in the control group).

### Neuropsychiatric events in LTMA users

Univariate and multivariate conditional logistic regression were used to estimate the risk of overall NPEs and three most frequent NPEs. Exposure to LTMAs was significantly associated with 1.58 times increased odds of NPEs in the unadjusted model (crude OR, 1.58; 95% CI 1.50–1.68). Similarly, for the three most frequent NPEs (sleep disorder, mood disorder and anxiety disorder), the odds of NPEs increased 1.49–1.53 times. (sleep disorder; 1.50, 95% CI 1.38―1.63; mood disorder; 1.49, 95% CI 1.37―1.63; anxiety disorder; 1.53, 95% CI 1.41―1.66). After controlling for covariates that were included in baseline characteristics in Table 1, the overall risk of NPEs in patients treated with LTMAs was also increased by nearly 70% compared to non-exposed patients (adjusted OR 1.67, 95% CI 1.58―1.78). Furthermore, LTMA treatment was associated with higher OR compared to no LTMA exposure for sleep disorder (1.54, 95% CI 1.42―1.68), mood disorder (1.65, 95% CI 1.51―1.81), and anxiety disorder, (1.63, 95% CI 1.50―1.77) (Table 2).

**Table 2:** Crude and adjusted odd ratios of leukotriene modifying agent exposure

	Overall NPEs	Sleep disorder	Mood disorder	Anxiety disorder
Crude OR (95% CI)	1.58 (1.50―1.68)	1.50 (1.38―1.63)	1.49 (1.37―1.63)	1.53 (1.41―1.66)
*p* value	< 0.01	< 0.01	< 0.01	< 0.01
Adjusted OR^a^ (95% CI)	1.67 (1.58―1.78)	1.54 (1.42―1.68)	1.65 (1.51―1.81)	1.63 (1.50―1.77)
*p* value	< 0.01	< 0.01	< 0.01	< 0.01

*Abbreviations*: *NPE* Neuropsychiatric Event, *OR* Odd Ratio, *CI* Confidence Interval

^a^ Adjusted by baseline characteristics in Table 1. Logistic regression was used in this analysis

### NPE risk by recency of exposure

The overall risk of NPEs and of the three NPEs was increased in all subgroups categorized by recency of exposure. In early period (< 60 days, group 1), the overall risk of NPEs was the highest in both the unadjusted and adjusted models. In the adjusted model, the overall risk of NPEs was almost twice than in LTMA non-exposed group (crude OR in group 1, 1.88, 95% CI 1.72―2.07; adjusted OR in group 1, 1.99, 95% CI 1.81―2.18). The overall risk of NPEs decreased gradually in both models. In the late period (> 120 days, group 3), the overall risk of NPEs was the lowest (crude OR in group 3, 1.38, 95% CI 1.27―1.50; adjusted OR in group 3; 1.46, 95% CI 1.34―1.59) (Table 3).

**Table 3:** Sub-group analysis, recency of exposure

	Overall NPEs		Sleep disorder		Mood disorder		Anxiety disorder	
	OR	CI	OR	CI	OR	CI	OR	CI
Group 1 (0–60 days)								
Crude OR	1.88	1.72–2.07	1.80	1.58–2.05	1.81	1.57–2.08	1.92	1.68–2.19
Adjusted^a^ OR	1.99	1.81–2.18	1.85	1.62–2.11	2.00	1.73–2.30	2.05	1.79–2.35
Group 2 (61–120 days)								
Crude OR	1.54	1.34–1.76	1.43	1.18–1.73	1.40	1.14–1.72	1.43	1.18–1.73
Adjusted^a^ OR	1.63	1.42–1.86	1.47	1.21–1.79	1.55	1.26–1.91	1.53	1.26–1.85
Group 3 (> 120 days)								
Crude OR	1.38	1.27–1.50	1.30	1.15–1.47	1.30	1.15–1.48	1.30	1.16–1.47
Adjusted^a^ OR	1.46	1.34–1.59	1.34	1.18–1.51	1.44	1.27–1.64	1.38	1.23–1.56

*Abbreviations*: *NPE* Neuropsychiatric Event, *OR* Odd Ratio, *CI* Confidence Interval

^a^ Adjusted by baseline characteristics in Table 2. Logistic regression was used in this analysis. All analyses have *p* value under 0.05

### NPE risk by duration of exposure

We classified patients who were exposed to LTMAs into three subgroups by duration of treatment. The risk of overall NPEs in patients treated for 31―120 days was the highest among the three subgroups.

The risk of overall NPEs was the lowest when patients used LTMA for more than 120 days (group 6) in both the unadjusted and adjusted models (crude OR, 1.51, 95% CI 1.31―1.75; adjusted OR, 1.60, 95% CI 1.38―1.85). In analyses of the three NPEs, the risk of every NPE had the highest OR in patients treated for 31―120 days in both unadjusted model and adjusted models (Table 4).

**Table 4:** Sub-group analysis, duration of drug use

	Overall NPEs		Sleep disorder		Mood disorder		Anxiety disorder	
	OR	CI	OR	CI	OR	CI	OR	CI
Group 4 (0–30 days)								
Crude OR	1.58	1.47–1.69	1.50	1.36–1.66	1.44	1.30–1.60	1.51	1.37–1.67
Adjusted^a^ OR	1.67	1.56–1.79	1.54	1.37–1.71	1.61	1.44–1.79	1.61	1.46–1.78
Group 5 (31–120 days)								
Crude OR	1.63	1.47–1.92	1.53	1.27–1.85	1.67	1.36–2.05	1.76	1.46–2.14
Adjusted^a^ OR	1.77	1.55–2.03	1.58	1.31–1.90	1.83	1.49–2.26	1.37	1.54–2.27
Group 6 (> 120 days)								
Crude OR	1.51	1.31–1.75	1.45	1.17–1.81	1.53	1.23–1.90	1.34	1.09–1.66
Adjusted^a^ OR	1.60	1.38–1.85	1.45	1.20–1.86	1.63	1.36–2.09	1.44	1.17–1.78

*Abbreviations*: *NPE* Neuropsychiatric Event, *OR* Odd Ratio, *CI* Confidence Interval

^a^ Adjusted by baseline characteristics in Table 2. Logistic regression was used in this analysis. All analyses have *p* value under 0.05

## Discussion

Our research was conducted to assess the risk of NPEs in elderly asthma patients who were treated with LTMAs. In this nested case-control study, asthma patients treated with LTMAs had 1.68 times higher risk of NPEs compared to asthma patients not treated with LTMAs, after controlling for socio-demographic factors and comorbidities. Among the risk of specific NPEs, the risk of anxiety disorder risk was the highest, but the difference was slight.

Our data on the association between NPEs and LTMA exposure are consistent with the results of several studies. Most of these previous studies were adverse drug reaction (ADR) studies using surveillance reports. A drug safety study that used data from the FDA Adverse Events Reporting System from 1999 to 2009 found that rates of reported completed suicides associated with montelukast increased substantially following warnings issued by the FDA [17]. Using a mixed-effects Poisson regression model, the authors found that empirical Bayes rates of patients treated with LTMAs were significantly greater than rates of patients treated with short-acting beta agonists. Haarman et al. [18] conducted an ADR study using the Netherlands Pharmacovigilance Center Lareb and VigiBase, the WHO Global database. In VigiBase, depression, insomnia and anxiety had higher reporting odds ratios (ROR) compared to other adverse drug reaction reports in the database (depression: ROR 6.93, 95% CI 6.54―7.36; insomnia; ROR 5.08, 95% CI 4.77―5.41; anxiety: ROR 5.11, 95% CI 4.79―5.41). In the Lareb database, insomnia and anxiety also had higher ROR compared to other drug adverse reaction reports (insomnia, ROR, 3.45; 95% CI; 2.05―5.81, anxiety, ROR, 2.79; 95% CI; 1.24―6.26), depression did not (ROR 1.91, 95% CI 0.79―4.62). There was a case series on neuropsychiatric ADRs such as nightmares, hallucinations and sleep walking that disappeared after discontinuation of montelukast [19]. Schumock et al. [20] conducted a nested case control study using an insurance claims database on suicide, which are a type of NPEs, and found that the risk of suicide attempt increased with LTMA users aged in 19–24 year in comparison with LTMA non users (adjusted OR: 5.15, 95% CI 1.16―22.86). Based on these studies, NPEs can be significantly increased with LTMA treatment in adults.

There were several studies that involved children. A nested case control study conducted by Glockler-Lauf et al. in Canada [21] showed that children with asthma who experienced a new-onset NPE had nearly double odds of having been prescribed montelukast in the year before the event (adjusted OR 1.91, 95% CI 1.15―3.18) [21] . Furthermore, according to Benard et al. [22], the relative risk of neuropsychiatric ADRs in children (as reported by parents) associated with montelukast versus inhaled corticosteroid was 12.0 (95% CI 1.6―90.2) [22].

On the other hand, in a nested case control study conducted by Schumock et al. [20], risk of suicide attempt in LTMA user had no significant correlation compared to patients who had never used LTMAs within age 5–11 years (adjusted OR 0.78, 95% CI 0.03―19.09) and 12–18 years (adjusted OR 0.47, 95% CI 0.20―1.09). A US observational study conducted by Mir et al. in 2015 [6] found that there was no consistent significant association between montelukast and NPEs. Any exposure to montelukast from past 30 days to past 365 days did not affect the OR of NPEs compared to non-exposed patients (past 30 days: adjusted OR, 1.02, 95% CI 0.82―1.26; past 90 days: adjusted OR, 1.00, 95% CI 0.82―1.22; past 180 days: adjusted OR, 0.99, 95% CI 0.83―1.19; past 365 days: adjusted OR, 0.96, 95% CI 0.80―1.14) [6]. These various studies have revealed inconsistent results about the risk of NPE in children after exposure to LTMAs. This could be due to the differences in patient population and changes of guidelines including LTMAs over time.

Although the molecular mechanism of association between NPEs and LTMAs remains unclear, there are several preclinical studies on the action of LTMAs in the brain and CNS. LTMAs (montelukast, pranlukast and zafirlukast) are anti-inflammatory drugs that specifically block the cysteinyl leukotriene type 1 (CysLT1) receptor [23]. By blocking this receptor, the drugs prevent the effects of CysLTs (LTC4, LTD4 and LTE4), which act to recruit inflammatory cells and increase vascular permeability [24]. Leukotrienes do not cross the blood-brain barrier (BBB) in any appreciable amounts; they are generated in brain tissue [25], and LTMAs, which can penetrate the BBB, reduce the neuro-inflammatory action of leukotrienes in the brain by blocking their receptor [26, 27]. This neuroprotective effect of LTMAs can ameliorate surgery-induced brain injury and protect against disruption of brain endothelial junction protein according to a murine study [28]. Also, knockdown of hippocampal CysLT1 receptor in mice prevents chronic mild stress-induced depressive-like behaviors and neuroinflammation by preventing the increases in hippocampal NF-κB, p65, IL-1β, and TNF-α [29]. While binding to the cysLT1 receptor, LTMAs can produce nitric oxides, which are toxic to brain tissue and damage it [30–32]; these data are consistent with an increase in NPE risk by LTMAs. Although FDA requires boxed warning and several studies of ADRs showed that LTMAs can increase the risk of NPEs, the results of preclinical studies, have been inconsistent. Thus, to obtain pharmacological evidence, more studies focused on the action of leukotrienes and LTMAs in CNS will be needed.

In our analysis of recency of exposure, the effect of increasing NPE risk was maintained until at least 1 year from taking LTMAs. In every NPE analysis, the risk in the early period (group 1) was the highest among the periods. Thus, we recommend to pay attention to ADRs, especially mood disorder and anxiety disorder, immediately after discontinuing LTMA treatment.

In regard to the duration of drug use, the risk of NPEs was highest in group 5 (31―120 days) and decreased after 120 days of treatment. We infer that if the treatment is continued over 120 days, BBB permeability of LTMAs may decrease slightly because of some kind of a defense mechanism for protecting the brain. However, although montelukast can reportedly decrease BBB permeability in rats [33], studies on BBB permeability of LTMAs in are scarce in human.

This study has several limitations. The NHIS database does not provide detailed information about asthma severity (e.g. how often reliever medication is used in a week, number of acute symptoms at night). Although some studies suggest that severe asthma or poor asthma control can increase the risk of NPEs such as depression and self-harm [21, 34–36], some factors such as FEV1 that reflect asthma severity could not be examined in our study. To make up for this, we performed 1:1 matching within asthma patients. Despite these limitations, this study also has several important strengths. First, we used the NHIS elderly cohort which includes 554,147 elderly patients. This large-scale patient database reflects clinical practice in real-world patients and enhances the statistical power in examining the outcome, thus enhancing the accuracy of the study. Second, this database guarantees the representativeness of the elderly in South Korea because it covers 99% of the Korean population. Because National Health Insurance provides lifetime coverage for South Korean citizens, the rate of dropout was low and the risk of selection bias was minimized. Third, to clarify the association between LTMAs and NPEs, we matched by propensity score that included 21 variables (5 socio-demographic variables, 16 comorbidity variables). Neuropsychiatric diseases have many confounders, which may hamper efforts to define the association between specific variables. By propensity score matching, we minimized the effect of confounders. Lastly, until now, there has been no observational study on the link between NPE risk and LTMA treatment in elderly asthma patients. Therefore, this research provides the first evidence for the need to care about elderly asthma patient’s neuropsychiatric ADRs.

## Conclusion

This study suggests that elderly asthma patients prescribed LTMAs had a higher risk of NPEs than patients who were not treated with LTMAs. The risk was highest within 60 days after taking LTMAs. The risk of all three specific NPEs (sleep disorder, mood disorder, anxiety disorder) was increased by LTMA treatment in every recency and duration of drug treatment. Therefore, clinicians should be aware of the potential risks of NPEs, especially in the early stages of LTMA treatment.

## Data Availability

The datasets used and/or analyzed during the current study are available from the corresponding author on reasonable request.

## Abbreviations

ADR: Adverse drug reaction
BBB: Blood brain barrier
CCI: Charlson comorbidity index
CI: Confidence interval
CysLT: Cysteinyl leukotriene
FDA: Food and drug administration in united states
HR: Hazard ratio
ICD-10: International classification of disease, tenth revision
ICS: Inhaled corticosteroid
LT: Leukotriene
LTMA: Leukotriene modifying agent
NHIS: National health insurance service
NPE: Neuropsychiatric event
OR: Odds ratio
ROR: Reporting odd ratio
HIV: Human Immunodeficiency Virus
